# Unusual *Cutibacterium acnes* splenic abscess with bacteremia in an immunocompetent man: phylotyping and clonal complex analysis

**DOI:** 10.1186/s12879-024-09467-x

**Published:** 2024-06-19

**Authors:** Angèle Roudeau, Stéphane Corvec, Beate Heym, Louise Ruffier d’Epenoux, Olivier Lidove, Valérie Zeller

**Affiliations:** 1 Department of Internal Medicine and Infectious Diseases, Groupe Hospitalier Diaconesses– Croix Saint-Simon, 125, rue d’Avron, Paris, 75020 France; 2 Service de Bactériologie et des Contrôles Microbiologiques, CHU Nantes, Université de Nantes, INSERM, INCIT U1302, Nantes, France; 3 Laboratoire des Centres de Santé et Hôpitaux d’Île-de-France, Groupe Hospitalier Diaconesses– Croix Saint-Simon, 125, rue d’Avron, Paris, France

**Keywords:** Abscess, Spleen, *Cutibacterium acnes*, Single-locus sequence typing, Phylotypes, Case report

## Abstract

**Background:**

*Cutibacterium acnes* is an anaerobic bacterium mostly implicated in cutaneous and body-implant infections. Splenic abscess is a rare entity and *C. acnes* abscesses have only exceptionally been reported. We describe a spontaneous splenic *C. acnes* abscess in an immunocompetent man with no predisposing factors or identified portal of entry. His isolates were subjected to single-locus sequence typing (SLST) to explore their genetic relatedness and better understand this rare infection.

**Case presentation:**

A splenic abscess was diagnosed on a computed-tomography scan in a 74-year-old man with chronic abdominal pain. No risk factor was identified. Abscess-drained pus and post-drainage blood cultures grew *C. acnes*. SLST of abscess and blood isolates showed that they belonged to the same *C. acnes* SLST type C1 found in normal skin and rarely in inflammatory skin disease. Specific virulence factors could not be identified.

**Conclusion:**

*C. acnes* abscesses are extremely rare and can develop in immunocompetent patients without an identifiable portal of entry. Molecular typing of clinical isolates can help confirm infection (versus contamination) and enables genetic background comparisons. Further research is needed to understand *C. acnes* tropism and virulence.

## Background

Splenic abscess develops extremely rarely (< 0.7% according to autopsy studies) [1] and *Cutibacterium acnes* abscesses have exceptionally been reported. Splenic abscesses are usually discovered in patients with endocarditis and considered to be of hematogenous origin [1]. Gram-positive cocci (*Staphylococcus aureus*, *Streptococcus viridans*) or Enterobacterales (*Klebsiella pneumoniae*) are classically responsible for these abscesses. Treatment requires splenectomy or percutaneous drainage and antibiotics. Reported mortality is 15–30% [1, 2].

*C. acnes* is a slow-growing, facultative, anaerobic, Gram-positive bacillus, mostly recovered as a saprophytic bacterium from human skin, the oral cavity, and gastrointestinal and genitourinary tracts. Its involvement in acne pathophysiology is well-known. Historically, *C. acnes* was considered a low-virulence microorganism. However, its pathogenic role, facilitated by its ability to produce biofilm, is now clearly established [3, 4].

We describe a spontaneous splenic *C. acnes* abscess, with bacteremia after abscess drainage, in an immunocompetent man with no predisposing factors or identified portal of entry, unlike the four cases reported in the literature [5–8]. The *C. acnes* isolates were subjected to single-locus sequence typing (SLST) to better understand this rare infection.

## Case presentation

**Fig. 1:**
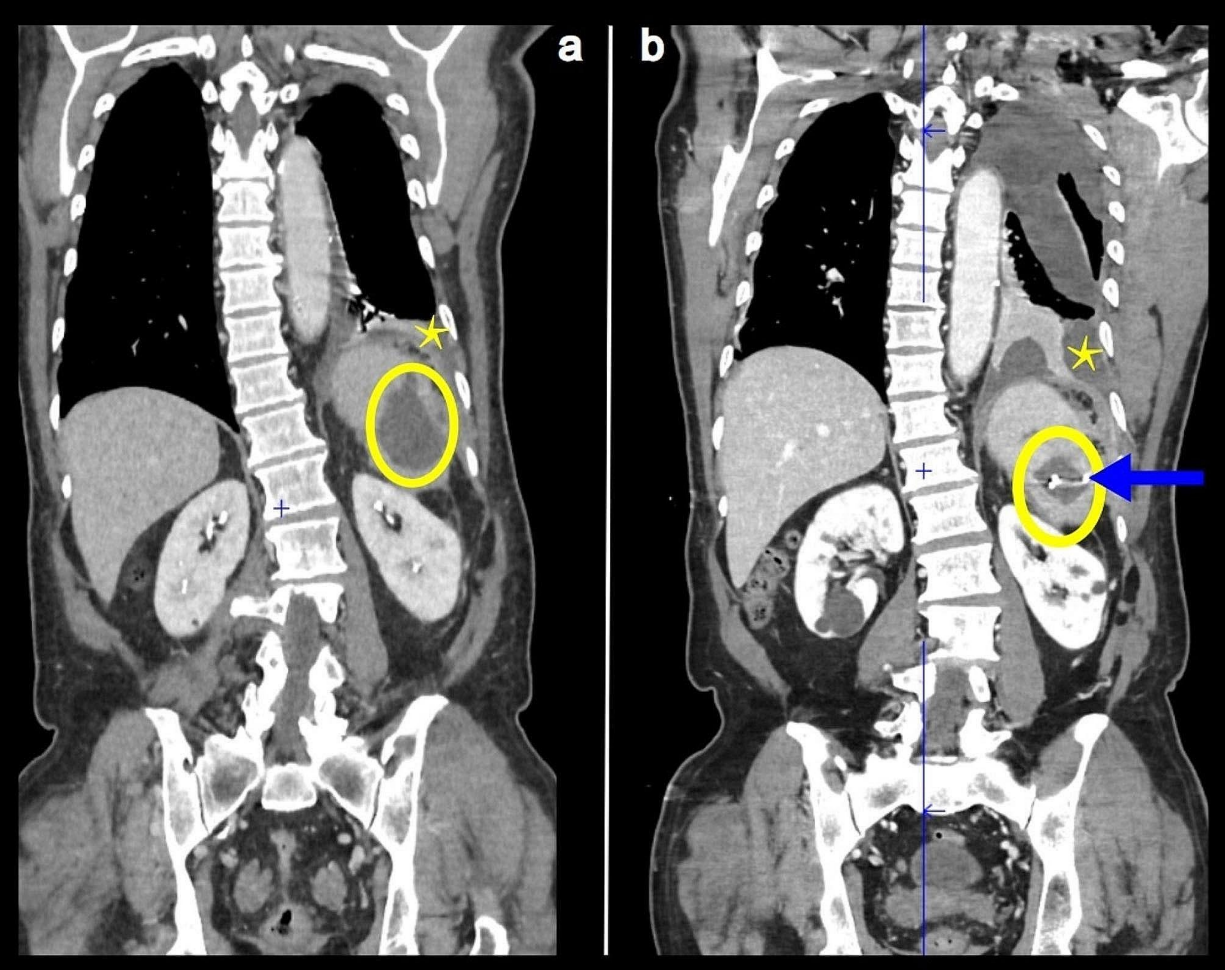
Thoracic-abdominal computed-tomography scan (a) before and (b) after abscess drainage. Splenic abscess (circle), pleural effusion (star) and drain (arrow)

A 74-year-old male was admitted to our Internal Medicine and Infectious Diseases Department in June 2022 for a second episode of abdominal pain, diarrhea, vomiting and fever, with 5-kg weight loss within 2 months. The first episode, 1 month earlier, had lasted 2 weeks. Abdominal computed-tomography (CT) scan showed a 7-cm splenic abscess (Fig. 1a). The gallbladder was normal. No blood cultures or other microbiological testing were performed. Admission findings were: weight 61 kg; afebrile without chills; complained of left hypochondrium and dorsal pains; stable hemodynamics and respiratory status; diminished breath sounds at the left lung base; no heart murmur, skin lesion or lymphadenopathy; soft but tender abdomen. Blood analyses showed: elevated C-reactive protein (CRP) 80 mg/L; normal neutrophil and eosinophil counts, creatinemia and liver enzymes.

Other remarkable history included: 2013, normal colonoscopy; 2009, normal coronarography; 2000, ischemic stroke; 1998, acute pulmonary edema. He reported no history of drug abuse.

Infective endocarditis was excluded in the absence of major and minor diagnostic criteria: transthoracic and transesophageal echographies showed no valvular lesion or suspicious image, but did reveal chronic heart failure. Three aerobic and anaerobic blood bottle cultures (FN plus bottle, bioMérieux, Inc Durham, NC27712, USA) of day-2, -6 and − 8 postadmission samples incubated for 120 h (5 days) were negative. He had no fever, cardiac murmur, predisposing heart condition enhancing the risk of infective endocarditis (no history of valvulopathy, previous endocarditis, prosthetic valve or other cardiac implants), other infectious sites in favor of septic embolism or immunological phenomena. Because of his African childhood, brucellosis, rickettsiosis, bartonellosis, echinococcosis and amoebiasis serologies were performed and were negative.

His clinical and biological parameters remained unchanged during week 1. On day-9 postadmission, before receiving any antibiotic therapy, he underwent CT-guided percutaneous spleen-abscess drainage. Drain placement was easy but the thick abscess pus was difficult to drain. Twenty-four hours later, he developed fever, chills, hypoxemia. A new CT scan showed enlargement of the left pleural effusion (Fig. 1b). Two blood cultures were initiated. Intravenous piperacillin–tazobactam was started but, upon appearance of an urticarial rash, switched immediately to ceftriaxone, amikacin, linezolid and metronidazole. Supplemental oxygen therapy was started (3 L/min) when breathing difficulty began before he was transferred to another hospital’s Thoracic Surgery Unit for left pleural drainage.

Direct examination of the splenic abscess pus found abundant Gram-positive bacilli. Numerous *C. acnes* colonies grew in pus cultures after 72 h of incubation and one of two anaerobic blood cultures, initiated just before starting antibiotics, also yielded *C. acnes* after 120 h of incubation. Isolate antibiotic susceptibilities were tested according to EUCAST guidelines using *Brucella*-agar plates containing 5% sheep blood, vitamin K1 (1 mg/L) and hemin (5 mg/L). A 1 McFarland-standard inoculum suspension was deposited on plates and incubated anaerobically. Gradient concentration strips were read after 48 h. The *C. acnes* strain was susceptible to amoxicillin (minimum inhibitory concentration (MIC) = 0.023 mg/L), ceftriaxone (MIC = 0.047 mg/L), rifampicin (MIC = 0.008 mg/L), vancomycin (MIC = 1 mg/L), daptomycin (MIC = 1.5 mg/L), clindamycin (MIC = 0.094 mg/L), doxycycline (MIC = 0.094 mg/L), moxifloxacin (MIC = 0.094 mg/L), levofloxacin (MIC = 0.25 mg/L) and linezolid (MIC = 0.094 mg/L) but resistant to metronidazole (MIC > 256 mg/L). Pleural effusion, collected 2 days after starting antibiotics, showed no growth. The differential cell count comprised 80% neutrophils; no absolute cell count was available.

Percutaneous spleen and pleural drainage lasted 10 days. The patient’s general condition progressively improved; he remained afebrile and supplemental oxygen was stopped after 1 week. His white blood count decreased from 18,600/mm^3^ to 8,800/mm^3^ and C-reactive protein (CRP) from 254 to 15 mg/L within 2 weeks. Antibiotic administration lasted 3 weeks. Intravenous (IV) ceftriaxone (2 g/day), oral linezolid (600 mg q12h) and oral metronidazole (500 mg q8h) were maintained for 10 days to treat the acalculous cholecystitis detected by abdominal echography. Treatment was then switched to oral clindamycin (600 mg, q8h) for 10 days to complete treatment of the *C. acnes* spleen abscess.

Immunosuppression searches remained negative: glycemia, protein electrophoresis, complete blood count, and immunoglobulin IgA, IgM and IgG rates were normal. Human immunodeficiency virus, hepatitis B and C virus serologies were negative.

The patient was discharged to home. One and a half years later, he was well, without any abdominal or infectious signs of relapse.

The *C. acnes* clinical isolates were sent to the *Cutibacterium* Species Reference Center (Nantes University Hospital) to investigate their genetic relatedness with molecular-typing methods including SLST [9, 10]. They belonged to SLST type C1, corresponding to phylotype IA_2_ and the University of Aarhus multilocus sequence typing clonal complex CC28 [11].

## Discussion and conclusion

To our knowledge, six cases of *C. acnes* splenic abscess have been published [5–8, 12, 13]; four were monomicrobial [5–8], like ours, and fully described, as summarized in Table 1. Another case in a retrospective study on splenic abscesses provided no further details [12], and the last, published > 40 years ago [13], reported a polymicrobial *S. aureus*-, *Clostridium* sp.- and *C. acnes*-containing abscess for which cross-contamination could not be excluded.

**Table 1:** Summary of case reports on monobacterial *C. acnes* splenic abscesses

Case [Ref] year	Age, yr, Sex	Risk factor(s)	Findings		Microbiology result(s), sample	Treatment	Outcome
			Clinical	Imaging			
1 [5] 1982	59, male	Diabetes, subcutaneous insulin, myocardial infarction	Left abdominal pain, nausea, vomiting, fever	11-cm splenic cystic deformity (ultrasonography & CT scan)	Positive blood-bottle cultures & spleen-pus culture	Splenectomy & IV penicillin	Survived
2 [6] 2013	64, male	Lymphocytic leukemia on IV chemotherapy	Fever of unknown origin	No anomaly on CT scan, high splenic uptake (FDG-PET)	Positive spleen specimen culture Sterile blood-bottle cultures	Splenectomy & levofloxacin	Survived
3 [7] 2017	64, female	Diabetes, IV-drug abuse	Nausea, vomiting, left abdominal pain	9-cm splenic hypodensity (CT scan)	Positive radio-guided spleen aspirate & spleen specimen cultures Sterile blood-bottle cultures	Metronidazole, ciprofloxacin & vancomycin, then splenectomy & clindamycin	Survived
4 [8] 2023	40, female	Tooth extraction	3-month history of fever, fatigue & left flank pain	Splenic abscess replacing almost the entire spleen parenchyma (CT scan)	Positive radio-guided spleen aspirate culture Sterile blood-bottle cultures	Metronidazole, ceftriaxone & splenectomy	Survived
5, This report 2023	72, male	None	2 episodes of nausea, vomiting, fever & left abdominal pain	7-cm splenic abscess (CT scan)	Positive radio-guided spleen aspirate & blood-bottle cultures	Percutaneous drainage; ceftriaxone, metronidazole & linezolid, then clindamycin	Survived

Abbreviations: CT, computed tomography; FDG-PET, ^18^F-fluorodeoxyglucose-positron emission tomography; IV, intravenous.

Our case, the fifth monomicrobial one, provides the first phylotyping of *C. acnes* abscess and blood isolates. It confirmed their genetic relatedness, better characterized them and excluded contamination.

Three previous cases [5, 6, 8] had usual clinical pictures, similar to our patient’s (Table 1). However, case 2 [7], was different; that heavily immunocompromised 64-year-old man was febrile without any gastrointestinal symptoms. His CT scan was normal. ^18^F-fluorodeoxyglucose–positron-emission tomography with high spleen uptake led to splenic abscess diagnosis.

Possible risk factors or portals of entry were sought for each of those rare infections. Because *C. acnes* is a human skin and buccal saprophytic bacterium, skin breaks were evoked for all of them: subcutaneous insulin [5], IV-drug abuse [6], IV chemotherapy [7] or tooth extractions [8]. However, those are common cutaneous breaks and splenic abscesses remain rare. The immunocompromised status of cases 1 and 2 (respectively, acute respiratory failure syndrome post-left myocardial infarction (8) or leukemia [7]) could explain their infections.

Major predisposing factors for *C. acnes* infection are previous surgeries and foreign-body implants, such as prosthetic heart valves, ventriculoperitoneal shunts, orthopedic implants and joint prostheses. *C. acnes* contamination most likely occurs during surgery. This bacterium has the capacity to adhere to the implant surface and persist through biofilm formation, with the latter being one of its major virulence factors [4]. Spontaneous *C. acnes* infections with bacteremia are very unusual. It is worth noting that, even though prosthesis *C. acnes* infections are now more common because of the higher number of prosthesis arthroplasties, none of the splenic abscess patients had a prosthetic device. Their infections are most probably attributable to contiguous cutaneous or mucosal contamination sources.

Our patient, unlike all previously reported cases, had no risk factor or determined source of *C. acnes* infection: no skin break (no IV or subcutaneous treatment, no recent surgery, no drug abuse, no dental work) and no prosthesis. He had not been hospitalized for more than 9 years. It is unlikely that contamination occurred at that time via IV infusion, for example, even if *C. acnes* is a slow-growing bacteria and the clone isolated from our patient is found in normal or chronically inflamed skin (see below). He was not immunosuppressed or immunocompromised and had neither endocarditis nor known splenic infarct. It seems improbable that acalculous cholecystitis caused the splenic abscess via the bloodstream, because *C. acnes* mainly inhabits deep skin layers. The patient had no right hypochondrial pain and his gallbladder was normal on the initial CT scan. It also seems unlikely that the CT-guided percutaneous abscess drainage infected the spleen: the puncture extracted large amounts of pus that, upon direct examination, contained numerous Gram-positive bacilli and cultures grew abundant *C. acnes* colonies.

Because we were unable to identify any risk factors for our patient, we investigated the idea that the risk was rather linked to the bacterium itself. Indeed, *C. acnes* expresses different antigens that could be involved in its pathogenicity [3]. The broad heterogenicity of *C. acnes* pathogenicity led to the development of several different accurate *C. acnes* phylogenic determination methods [5]. *C. acnes* strains can be divided into at least six main phylotypes (IA_1_, IA_2_, IB, IC, II and III). SLST determined different lineages and divided them into at least 194 phenotypes [10], which are increasingly linked to different pathogenic capabilities [14]. Our patient’s three isolates (one from blood bottle cultures and two from splenic pus) were subjected to molecular typing including SLST, phylotyping and multilocus sequence typing. All three belonged to the same clone: SLST type C1, phylotype IA_2_ and clonal complex CC28. Notably, this cluster has been found in normal skin and rarely in chronic inflammatory skin diseases, e.g., acne [3, 15]. It seems to have high biofilm-formation capacity and strong adhesive properties [14]. Despite genomic investigations, it has proven difficult to identify specific virulence factors [9, 15] for this *C. acnes* subtype. These results suggest that the skin was the most likely source of infection. The bacteria might have spread either via the blood or locally from a nearby source. The clinical picture and the absence of an identified portal of entry could not confirm that hypothesis, despite our investigations.

Our patient underwent CT-controlled percutaneous drainage, which was developed for splenic abscesses in the 1990’s [16]. The other *C. acnes* splenic abscesses were treated with splenectomy [5–8] (**Table 1**). Percutaneous drainage was chosen because the patient was pauci-symptomatic and his chronic heart failure put him at high risk for surgery. The initial drainage was difficult because of pus thickness and led to sepsis 24 h later, and worsening of the pleural effusion requiring prolonged drainage. Finally, 10 days of percutaneous drainage and 3 weeks of antibiotics eradicated his infection.

*C. acnes* is naturally metronidazole- and fosfomycin-resistant, and susceptible to a wide range of antibiotics (β-lactam, clindamycin, rifampicin, vancomycin, daptomycin and fluoroquinolones). No consensus exists for first-choice and alternative antibiotic regimens, which remain controversial [3]: amoxicillin, clindamycin or fluoroquinolones. Available data derive from implant-associated infection [4]. Large-scale studies on homogeneous populations are rare, the antibiotic regimens used to treat these infections are heterogeneous and no recommendations based on a high level of evidence can be devised. The antibiotic regimens prescribed for all reported *C. acnes* splenic abscesses differed (**Table 1**). Our patient initially received a broad-spectrum antibiotic combination to cover other gastrointestinal bacteria and acalculous cholecystitis, before being narrowed to clindamycin to treat the *C. acnes* spleen abscess. Clindamycin was chosen because of its good activity, bioavailability and tissue diffusion. Amoxicillin is another highly active agent.

## Conclusion

This *C. acnes* splenic abscess developed in a patient without any potentially responsible comorbidity. It illustrates that immunocompetent patients—without an evident skin break or other portal of entry—can develop this extremely rare infection. Molecular typing of clinical isolates can help confirm infection (versus contamination) and enable genetic background comparisons. Further research, especially on virulence factors, is still needed to understand this unusual tropism.

## Data Availability

All data analyzed during this study are included in this article.

## Abbreviations

CRP: C-reactive protein
CT scan: Computed tomography
IV: Intravenous
MIC: Minimum inhibitory concentration
SLST: Single-locus sequence typing
